# On causality and mechanisms in medical education research: an example of path analysis

**DOI:** 10.1007/s40037-015-0174-z

**Published:** 2015-04-09

**Authors:** Jimmie Leppink

**Affiliations:** School of Health Professions Education, Department of Educational Development and Research, Maastricht University, PO Box 616, 6200 MD Maastricht, The Netherlands

**Keywords:** Experimental research, Causality, Path analysis, Treatment effect, Mediation

## Abstract

Studies in medical education can serve a variety of purposes. Studies that have a predominantly quantitative orientation may focus on estimating relations between variables, on estimating effects of one or more variables on some other variable(s), or on series of causal relations or mechanisms. Which is the focus of a particular study depends on the theoretical framework and research questions of that study. However, theory is of fundamental importance to medical education research, and studies focusing on series of causal relations or mechanisms can contribute greatly to the advancement of medical education research. This paper presents the potential benefits which result from adopting a path analysis perspective on the estimation of causal relations and conceptualization of mechanisms in medical education research.

## Introduction

Studies in medical education can serve a variety of purposes. Studies that have a mainly quantitative orientation may focus on estimating relations between variables, on estimating effects of one or more variables on some other variable(s), or on series of causal relations or mechanisms. Which is the focus of a particular study depends on the theoretical framework and research questions of that study. However, theory is of fundamental importance to medical education research, and studies focusing on series of causal relations or mechanisms can contribute greatly to the advancement of medical education research. This paper presents the potential benefits which result from adopting a path analysis [1–7] perspective on the estimation of causal relations and conceptualization of mechanisms in medical education research. This is done through a comparison of this frequently overlooked method with two frequently encountered methods—one-way analysis of variance (ANOVA) and analysis of covariance (ANCOVA) [8]—in a hypothetical study with simulated data. More specifically, the hypothetical study is about a randomized controlled experiment. The choice of this specific context should neither be interpreted as a statement that path analysis is a tool to be exclusively used in the context of randomized controlled experiments nor should it be taken as a message that path analysis is by definition necessary or superior in such a context. If the purpose of an experiment is to estimate effects, there may be no need for path analysis. However, if there is an interest in understanding causal relations or mechanisms in this context, path analysis is to be preferred.

The reasons for choosing the context of a randomized controlled experiment are threefold. Firstly, medical education research increasingly makes use of randomized controlled experiments and the domain is doing a good job in designing good experiments and avoiding common pitfalls [9]. Secondly, a randomized controlled experiment provides a fairly easy context for making the comparison between path analysis and the other two methods. Thirdly, there appears to be a lack of awareness among researchers that an experimental setup and timing of measuring variables of interest in a randomized controlled experiment have implications for data analysis when there is an interest in causal relations or mechanisms [10].

For the sake of simplicity, the example is one in which there is no significant moderation or interaction between treatment and self-study time, that is: the difference between experimental treatment condition and control condition in average performance does not differ significantly across the range of self-study time or, in other words, the effect of self-study time on performance is more or less the same for the two conditions. Although it is possible to have moderation (i.e., interaction) and mediation going on at the same time, the aim of this paper is to increase awareness among medical education researchers of the differences in the three approaches discussed (i.e., ANOVA, ANCOVA, and path analysis) and of the danger of ‘controlling’ for a mediator variable. For those who are interested in reading more about modelling mediation and moderation, see the classical paper on the moderator-mediator variable distinction by Baron and Kenny [1] as a starter, and find more recent developments in work by Edwards and Lambert [3], Frazier and colleagues [4], Judd and colleagues [5], Kraemer and colleagues [6], and Muller and colleagues [7]. Further, this example does not involve latent variables. This is because explaining the concept of latent variables for structural equation modelling (or other latent variable models) and concepts and assumptions with it could easily fill another paper in order to serve a wide audience. Finally, the example also refrains from situations in which participants collaborate in learning groups during a course or a situation in which participants are measured repeatedly on the same kind of performance. The latter would require an explanation of multilevel analysis, to then fit path analysis in a multilevel context. The interested reader is referred to other sources for a primer on multilevel analysis [11–13] and multilevel analysis in medical education research [14], introductory texts in structural equation modelling with latent variables [15–17], and for the more advanced, multilevel structural equation modelling [11, 16].

### The hypothetical study

Suppose, a researcher is interested in the effect of a new instructional method on self-study time and on test performance right after an anatomy course, and decides to conduct a randomized controlled experiment.

**Fig. 1:**
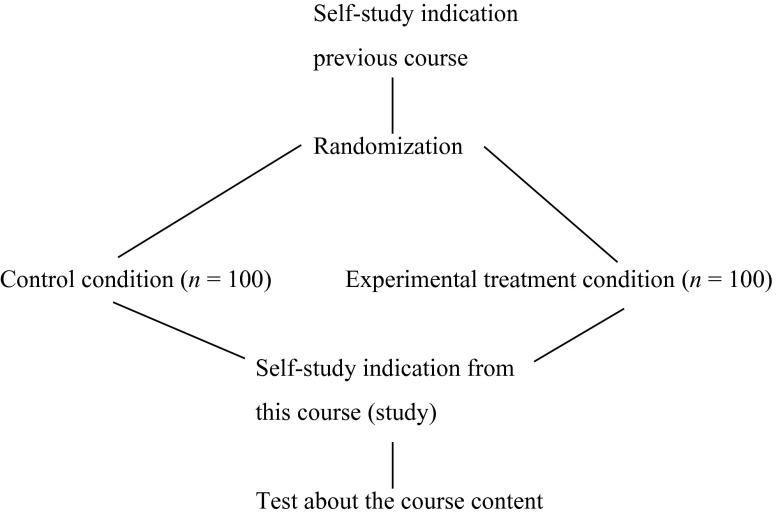
Study design used as example in this paper

The researcher decides to randomly allocate 200 students currently enrolled in her course to either the new instructional method (i.e., experimental treatment condition) or the traditional method (i.e., control condition). Both groups attend the course in the same seven-week period, study exactly the same materials, and perform exactly the same test which results in a score varying from 0 (minimum) to 100 (maximum). The only difference between the two groups lies in the information students receive about the way they are going to be assessed right after the course. This is done because the researcher is interested in the pre-assessment learning effect [18] that this new instructional method has on test performance after the course. That is, the researcher expects that the new way of informing students about the assessment (i.e., experimental treatment condition) will stimulate them to engage in a more in-depth self-study and consequently demonstrate better test performance compared with the traditional way of informing students (i.e., control condition), the method that has been standard practice in the course thus far. Prior to taking the test about the course content, students complete a course evaluation form, which includes the question how many hours the (individual) student spent on self-study over the 7 weeks together. Students’ responses to that question vary from 24 to 86 h. Finally, the researcher also has access to students’ response to the latter question for the previous seven-week course, on basic medical science, which was taught by a colleague. Students’ responses to that question vary from 18 to 44 h. The researcher decides to use that information for a randomization check in her experiment. This test indicates to what extent randomly allocating students to experimental or control condition resulted in comparable groups. Figure 1 visualizes the study design described.

The researcher wants to use the outcomes of the experiment to decide whether the new instructional method should be used in subsequent cohorts of students taking the course. Further, as the course is also taught in a similar form in medical faculties in other institutions, the researcher intends to submit a paper presenting the outcomes of the experiments to a peer-reviewed journal. The following two hypotheses are to be tested and reported in the paper:

- Hypothesis 1 (*H1*), *effect of treatment on self-study time*: students in the experimental treatment condition spend on average more time on self-study than their peers in the control condition; and
- Hypothesis 2 (*H2*), *effect of treatment on test performance*: students in the experimental treatment condition perform on average better on the test than their peers in the control condition.

### Approach

While testing *H1* comes down to a one-way analysis of variance (ANOVA) or two samples *t*-test with self-study time as dependent variable and treatment as independent variable, there are three approaches to testing *H2*:

- Approach (*1*), *ignore self-study time*: one-way ANOVA or two samples *t*-test with test performance as dependent variable and treatment as independent variable;
- Approach (*2*), *self-study time as covariate*: analysis of covariance (ANCOVA) with test performance as dependent variable, treatment as independent variable, and self-study time as covariate; and
- Approach (*3*), *self-study time as mediator*: path analysis with test performance as dependent variable, and treatment as independent variable as mediator.

Thus, the three approaches to testing *H2* differ in the role of self-study time in the analysis of the effect of treatment on performance.

## Method

Data from this design were simulated using SPSS v21; an overview of the simulation procedure is available from the author.

The advantage of a simulation study is that the outcomes of the study are known and as such it allows comparison of the strengths and weaknesses of various methods of analysis, here the three approaches to testing *H2*. All analyses were performed in Mplus v7.2 but could be done in other programmes as well (e.g., R, STATA, SAS, LISREL, AMOS, and SPSS).

**Fig. 2:**
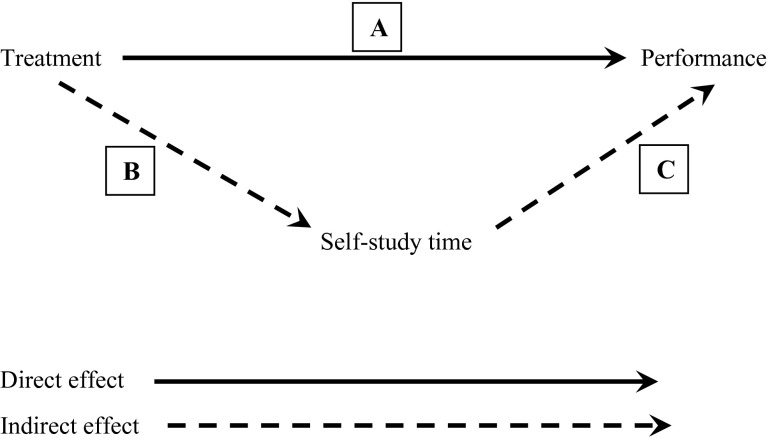
Decomposition of the effect of treatment on performance

The difference between the three approaches can be understood starting from Fig. 2, which presents the effect of treatment on performance as a sum of that part of the effect of treatment on performance that goes through (i.e., is mediated by) self-study time and that part of the effect of treatment of performance that is independent of self-study time.

That part of the effect of treatment on performance that is mediated by self-study time is also referred to as the *indirect effect* of treatment on performance, while that part of the effect of treatment on performance that is independent of self-study time is also called the *direct effect*. The *total effect* of treatment on performance equals (the coefficient of) A plus the product of (the coefficients of) B and C (i.e., A + BC).

### Approach (1): ignore self-study time

The one-way approach makes *no distinction between direct and indirect effect*; it is simply a *test on the total effect* of treatment on performance. The rationale behind this approach is that *H2* (i.e., students in the experimental treatment condition perform on average higher on the test than their peers in the control condition) in fact pertains to the *total effect*, which is the sum of direct and indirect effect. Proponents of this approach may agree that this total effect of treatment on performance can at least partly be explained by differences in self-study time. However, random allocation to experimental treatment and control condition usually results in comparable groups in terms of willingness to spend more time on self-study (especially in the case when sample sizes are *n* = 100, as in the example in this paper). Since self-study time during the course is measured *after* the start of the experimental manipulation, differences in average self-study time between the two conditions can be attributed to treatment. This is why, if self-study time influences performance, increased performance through a higher average self-study time in one condition versus the other is in fact part of the treatment effect. If one is interested *only* in the effect of treatment on performance and not also in the extent to which that treatment effect is mediated by self-study time, this one-way (i.e., ignore self-study time) provides a valid approach to testing *H2*. This is the case because we are dealing with a randomized controlled experiment in which differences in self-study time during the experiment can be attributed to treatment. In quasi-experimental studies, in which no random allocation to conditions takes place (i.e., many ‘in-vivo’ field studies in medical education are quasi-experimental) and therefore causal conclusions are only arguably allowed, potential confounding by self-study time needs to be controlled for.

### Approach (2): self-study time as covariate

While the one-way approach compares the experimental treatment and control condition in terms of A plus BC, the self-study time as covariate approach compares the two conditions only in terms of A. The causal relation between treatment and self-study time is ignored and with it the indirect effect of treatment (through self-study time) on performance. In fact, this approach assumes self-study time to be a confounding variable. In other words, in this approach, self-study time is assumed to be *correlated* with (instead of causally influenced by) treatment; this meanwhile affects performance, and therefore ‘needs to be controlled for’ when estimating the treatment effect. This distinction between correlation and causation is of great importance for science and forms a contrast between this approach and the other two approaches. There are two situations in which this approach is valid. Firstly, in quasi-experimental studies, in which no random allocation to conditions takes place and therefore causal conclusions are only arguably allowed, potential confounding by self-study time needs to be controlled for. Secondly, if—in quasi-experimental or experimental studies—treatment and self-study time turn out to be more or less unrelated but self-study time does affect performance, including self-study time as a covariate increases the statistical power for testing the effect of treatment on performance [10]. However, if in an experiment such as the one in this example self-study time is causally influenced by treatment and it affects performance, the self-study time as covariate approach reduces the treatment effect of A plus BC to just A.

### Approach (3): self-study time as mediator

**Fig. 3:**
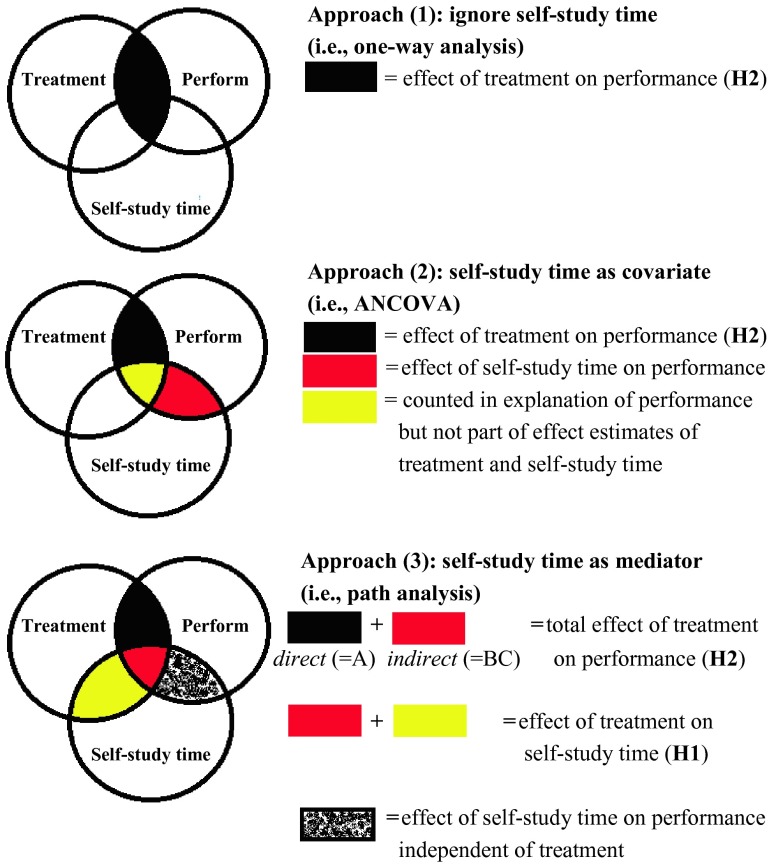
Graphical comparison of the three approaches

The self-study time as mediator or path analysis approach uses all information displayed in Fig. 2, that is: A, B, and C are recognized and estimated as three causal paths. In accordance with the self-study time as covariate approach, path A represents the direct effect of treatment on performance, that is: the effect of treatment on performance independent of self-study time. Further, when summing A (direct effect) and BC (indirect effect), we obtain the total effect that we get in the one-way approach. Finally, path B is what the researcher intends to test with *H1*. In the other two approaches, we would need to run a separate test for *H1*, whereas in the path analysis approach *H1* is automatically included. In other words, the self-study time as mediator approach encompasses the other two approaches and enables simultaneous testing of *H1* and *H2* in one (path) model. Note that in quasi-experimental studies, this approach might not make much sense because causality may be left in the middle. How do we know, when using pre-existing groups instead of groups resulting from random allocation, that treatment causally influences self-study time and not the other way around or that both just co-vary simultaneously? In the context of an experiment as the example in this paper, however, the path analysis approach appears the best approach if treatment significantly affects self-study time. If treatment does not result in significant changes in self-study time we can also go with the self-study time as covariate approach, for that approach provides outcomes of paths A and C as well. In that case, there is no significant indirect effect, and treatment and self-study time can be regarded as affecting performance more or less independently. Figure 3 graphically compares the three approaches in Venn diagrams. These diagrams help to understand the findings presented in the next section.

## Results

In the control condition, students reported on average 29.69 (*SD* = 4.964, skew = 0.163, kurtosis = − 0.216) hours of self-study in the previous course (i.e., prior to the experiment), on average 50.42 (*SD* = 9.131, skew = − 0.316, kurtosis = 0.173) hours of self-study during the experiment (course), and the average test performance was 50.79 (*SD* = 11.130, skew = 0.346, kurtosis = − 0.275). In the experimental treatment condition, students reported on average 29.94 (*SD* = 5.103, skew = 0.074, kurtosis = − 0.303) hours of self-study in the previous course (i.e., prior to the experiment), on average 55.43 (*SD* = 9.249, skew = − 0.016, kurtosis = 1.120) hours of self-study during the experiment (course), and the average performance was 55.29 (*SD* = 10.883, skew = 0.149, kurtosis = − 0.059).

The difference between groups in average self-study time in the previous course is close to zero and not statistically significant: we find a standardized regression coefficient (*β*) of 0.025 (*SE* = 0.071), *t* = 0.353, *p* = 0.724. Standardized regression coefficients of around 0.10, 0.25, and 0.40 represent small, medium, and large effects, respectively [19]. In other words, we are dealing with a very small effect (if any) that is not statistically significant at the conventional *α* = 0.05 significance level. This provides support for the assumption that the researcher succeeded in creating two comparable groups through randomization.

For *H1* (i.e., path B in Fig. 2), we find *β* = 0.264 (*SE* = 0.066), *t* = 4.017, *p* < 0.001. In other words, the effect of treatment on self-study time is of medium size. It is positive because average self-study time was higher in the experimental treatment condition and we apply conventional coding of ‘0’ for the control condition and ‘1’ for the experimental treatment condition (i.e., dummy coding with ‘1’ representing the experimental treatment condition). If we reversed the 0/1 condition (i.e., ‘1’ for control condition), we would obtain − 0.264 as standardized regression coefficient.

The standardized regression coefficient of 0.264 is statistically significant at the conventional *α* = 0.05 significance level, thus we reject the null hypothesis that the treatment does not affect self-study time, and we have significant support for *H1*. As we have discussed, and will see in the following, this has implications for the validity of self-study time as covariate approach. We now discuss each of the three approaches in the light of the outcomes for *H2*.

### Approach (1): the easy way

A one-way test for *H2* (see first diagram in Fig. 3) reveals *β* = 0.201 (*SE* = 0.068), *t* = 2.966, *p* = 0.003. If one is not interested in the extent to which this treatment effect is mediated by self-study time, the analysis can stop here: one reports the two *β*-values for *H1* and *H2*, respectively, and the discussion section follows. This is the case because we are dealing with a randomized experiment in which, contrary to what would be the case in quasi-experimental studies, differences in self-study time during the experiment can be attributed to treatment.

### Approach (2): ‘controlling’ for self-study time

For the ‘treatment’ effect on performance, which is in fact the direct effect (i.e., path A in Fig. 2), the ANCOVA approach yields *β* = 0.093 (*SE* = 0.066), *t* = 1.426, *p* = 0.154. For the unique effect of self-study time on performance (see second diagram in Fig. 3), we find *β* = 0.408 (*SE* = 0.061), *t* = 6.740, *p* < 0.001 (i.e., path C in Fig. 2). The standardized residual variance is 0.805, which is the complement of the proportion of variation in performance explained by the combination of treatment and self-study time. Thus, about 19.5 % of the variation in performance can be explained by the combination of treatment and self-study time. As we see, the *β*-value of the treatment effect is considerably smaller than in the one-way approach. This is because the indirect effect—which is positive (i.e., positive effect of treatment on self-study time and positive effect of self-study time on performance)—is ignored. In other words, we should see in the next paragraph that the indirect effect explains the difference between *β* = 0.201 (one-way approach) and *β* = 0.093 (this approach).

### Approach (3): path analysis

The path analysis returns values we have seen before: *β* = 0.093 for path A in Fig. 2, *β* = 0.264 for path B in Fig. 2 (i.e., *H1*), and 0.408 for path C in Fig. 2. We have defined the total effect of treatment on performance as the sum of A and (product) BC:

$${\rm{A}}\, + \,({\rm{B }} \times {\rm{ C}})\, = \,0.093\, + \,\left( {0.264{\rm{ }} \times {\rm{ }}0.408} \right){\rm{ }} \approx {\rm{ }}0.201$$

In other words, we obtain the same value as in the one-way approach (differences at the fourth decimal point are due to a round-off error), only now we obtain outcomes *H1* and *H2* simultaneously, along with an estimate of the effect of self-study time on performance independent of treatment (see the third diagram in Fig. 3), in one single model.

Further, the standardized residual variance is the same as in the ANCOVA approach: 0.805 (compare the second and third diagram in Fig. 3; the uncoloured surface in the ‘performance’ circle is the same). Thus, we reach the same conclusion with regard to the variation in performance that is explained by the combination of treatment and self-study time. However, in the ANCOVA approach, this combination is conceptualized as (1) the effect of treatment independent of self-study time (i.e., direct effect or path A in Fig. 2, the black area in the second diagram in Fig. 3), (2) the effect of self-study time independent of treatment (i.e., path C in Fig. 2, the red area in the second diagram in Fig. 3), and (3) the overlap (i.e., correlation) between treatment and self-study time which is counted in the 19.5 % of the explained variation in performance but which is allocated to neither treatment nor self-study time effect estimate (i.e., the yellow area in the second diagram in Fig. 3). In the path analysis approach, the latter is conceptualized and estimated as the *indirect effect* of treatment, that is: that part of the treatment effect that is mediated by self-study time.

In other words, the path analysis approach unites the better of the other two approaches and adds something extra. Firstly, as in the one-way approach (but contrary to the ANCOVA approach), a correct estimate of the overall effect of treatment on performance is provided. Secondly, as in the ANCOVA approach (but contrary to the one-way approach), we can estimate how much of the variation in performance can be explained by the combination of treatment and self-study time. Thirdly, as path analysis—contrary to the other two approaches—includes all paths (and as such enables simultaneous testing of *H1* and *H2*), we can come to understand more about a particular mechanism or process (i.e., through self-study time) in the treatment effect. Finally, for the sake of simplicity of the example, we have discussed an example with one mediator. It is of course possible that multiple variables (e.g., mental effort, motivation to learn) are measured after treatment but before performance in a randomized controlled experiment, and in that case path analysis can help us to learn more about more than one process in the treatment effect.

## Discussion

While the path analysis approach (in which self-study time is treated as mediator) arguably makes more sense than the ANCOVA approach (in which self-study time is treated as a covariate) in quasi-experimental studies, it is generally to be preferred in randomized controlled experiments in which treatment affects self-study time. Of course, self-study time is just one example of a potential mediator. In fact, any variable measured *after* the start of treatment—mental effort, motivation to learn, compliance to treatment, you name it—is a mediator if it is affected by treatment and in its turn affects a dependent variable measured at a later point in time (in our example: performance). Although the simple one-way approach is sufficient in such cases if one is interested only in the total treatment effect but not in any kind of mediation, mediation analysis is crucial for theory development, especially for theories regarding *mechanisms* or *processes* [1–2]. For instance, in the example in this paper, if—based on real instead of simulated data—the researcher were to conclude that the treatment effect is mainly if not exclusively (i.e., the direct effect is not statistically significant) a matter of on average more self-study time, and a subsequent study—which should probably also include *qualitative* methods—might zoom in more on whether this increase in self-study time reflected mainly repeated practice of the same content or perhaps more in-depth study of particular content. The one-way (i.e., ANOVA) approach, on the contrary, leaves us blind with regard to the role of self-study time in the (mediation of the) treatment effect, and the ANCOVA approach focuses only on the unique contribution of either of treatment and self-study time but not on the mediation (i.e., indirect effect) part (compare the second and third diagram in Fig. 3).

Contrary to the frequently encountered ANOVA and ANCOVA, path analysis appears to be overlooked by many medical education researchers. However, understanding mechanisms or processes is of vital importance to the domain, and path analysis constitutes a useful way to gain that understanding. It is to be hoped that more researchers in medical education will use path analysis for this purpose.

## Essentials

Bullet points summarizing the key messages of the article:

- Path analysis is a useful yet often overlooked tool to study causal relationships and mechanisms that can as such contribute greatly to the further development of medical education theory.
- In randomized controlled experiments, variables measured after the start of treatment should generally be treated as potential mediators, not as confounders, of treatment effects.
- In randomized controlled experiments, including a mediator of treatment effects in analysis of covariance (ANCOVA), to treat it as a covariate, results in incorrect estimates of effects of treatment on response variables of interest.
- While in quasi-experimental studies, ANCOVA may make more sense than path analysis (due to a lack of ability to establish causality), in randomized controlled experiments, path analysis is generally to be preferred.

### Source(s) of support in the form of grants

None.
